# A Machine Learning Model to Predict Risperidone Active Moiety Concentration Based on Initial Therapeutic Drug Monitoring

**DOI:** 10.3389/fpsyt.2021.711868

**Published:** 2021-11-18

**Authors:** Wei Guo, Ze Yu, Ya Gao, Xiaoqian Lan, Yannan Zang, Peng Yu, Zeyuan Wang, Wenzhuo Sun, Xin Hao, Fei Gao

**Affiliations:** ^1^Beijing Key Laboratory of Mental Disorders, The National Clinical Research Center for Mental Disorders, Beijing Anding Hospital, Capital Medical University, Beijing, China; ^2^Advanced Innovation Center for Human Brain Protection, Capital Medical University, Beijing, China; ^3^Beijing Medicinovo Technology Co. Ltd., Beijing, China; ^4^Lugouqiao Community Health Service Center, Beijing, China; ^5^School of Computer Science, The University of Sydney, Sydney, NSW, Australia; ^6^Xi'an Jiaotong-liverpool University, Suzhou, China; ^7^Dalian Medicinovo Technology Co. Ltd., Dalian, China

**Keywords:** risperidone, active moiety, XGBoost, machine learning, prediction model

## Abstract

Risperidone is an efficacious second-generation antipsychotic (SGA) to treat a wide spectrum of psychiatric diseases, whereas its active moiety (risperidone and 9-hydroxyrisperidone) concentration without a therapeutic reference range may increase the risk of adverse drug reactions. We aimed to establish a prediction model of risperidone active moiety concentration in the next therapeutic drug monitoring (TDM) based on the initial TDM information using machine learning methods. A total of 983 patients treated with risperidone between May 2017 and May 2018 in Beijing Anding Hospital were collected as the data set. Sixteen predictors (the initial TDM value, dosage, age, WBC, PLT, BUN, weight, BMI, prolactin, ALT, MECT, Cr, AST, Ccr, TDM interval, and RBC) were screened from 26 variables through univariate analysis (*p* < 0.05) and XGBoost (importance score >0). Ten algorithms (XGBoost, LightGBM, CatBoost, AdaBoost, Random Forest, support vector machine, lasso regression, ridge regression, linear regression, and k-nearest neighbor) compared the model performance, and ultimately, XGBoost was chosen to establish the prediction model. A cohort of 210 patients treated with risperidone between March 1, 2019, and May 31, 2019, in Beijing Anding Hospital was used to validate the model. Finally, the prediction model was evaluated, obtaining *R*^2^ (0.512 in test cohort; 0.374 in validation cohort), MAE (10.97 in test cohort; 12.07 in validation cohort), MSE (198.55 in test cohort; 324.15 in validation cohort), RMSE (14.09 in test cohort; 18.00 in validation cohort), and accuracy of the predicted TDM within ±30% of the actual TDM (54.82% in test cohort; 60.95% in validation cohort). The prediction model has promising performance to facilitate rational risperidone regimen on an individualized level and provide reference for other antipsychotic drugs' risk prediction.

## Background

As one of the representative atypical antipsychotic drugs, risperidone is a benzisoxazole derivative with potent serotonin (5-hydroxytryptamine) antagonism and moderate dopamine (D2) receptor-blocking effects (1, 2). Hitherto, risperidone has been proven to be one of the most efficacious second-generation antipsychotics (SGAs) to treat a wide spectrum of psychiatric diseases, especially for positive and negative schizophrenic symptoms (3–6). Besides this, according to the Food and Drug Administration and European Medicines Agency, risperidone has various indications, such as irritability associated with autistic disorder, manic episodes associated with bipolar disorder, and persistent aggression in patients with mental retardation or Alzheimer's dementia (3, 7). It also shows efficacy in treating disruptive behavior, tic, and attention-deficit/ hyperactivity disorders (8). The main pathway of risperidone metabolism is 9-hydroxylation catalyzed by the cytochrome P450 2D6 (CYP2D6) in the liver, producing the primary active metabolite 9-hydroxyrisperidone (4, 8, 9). The combined risperidone and 9-hydroxyrisperidone serum concentration is termed as “active moiety,” which displays association with adverse drug reactions (ADRs) and clinical treatment effects (10, 11). Short-term usage of risperidone could lead to some rapid occurrence of ADRs, such as nausea, vomiting, and excessive sedation. In addition, some long-term ADRs induced by risperidone were also received with concerns, such as obesity, insulin resistance, leptin resistance, and hyperuricemia, depending on risperidone dosage or treating period (12–15). Therapeutic drug monitoring (TDM) is a vital method to ensure individualized antipsychotic regimen with low ADR risks and higher treatment efficacy (8, 16). In accordance with the Arbeitsgemeinschaft für Neuropsychopharmakologieund Pharmakopsychiatrie (AGNP) consensus guidelines, the recommended therapeutic reference range is 20~60 ng/ml for the risperidone active moiety (4, 9).

Machine learning is a rising technique applied in medical research recently. It not only can process high-volume data to identify important factors, but also captures nonlinear variable relations to achieve high accuracy in predicting clinical outcomes, which is an indispensable approach for solving complex issues in different fields, such as image interpretation and cancer prognosis (17). For instance, machine learning algorithms are applied in 24 studies about bipolar disorder, which are proven to be useful in implementing risk assessment and early detecting latent bipolar disorder patients (18). EXtreme Gradient Boosting (XGBoost) is one of the competent classifier construction algorithms seen in various classification or regression studies with promising prediction results, such as the risk prediction model for type 2 diabetes (19). In short, a machine learning technique is adaptable in diagnosis, individualized treatment, and prognosis orientation, especially for multifactorial diseases.

The TDM value prediction of a specific drug remains a challenge in clinical practice. Our objective was to establish a prediction model of risperidone active moiety concentration in the next TDM based on the initial TDM information using machine learning methods. XGBoost was proposed as the major method in this study to deal with mass data from the real world to find the influencing factors for risperidone TDM value and develop a prediction model.

## Methods

### Participants and Study Design

A retrospective analysis of patients with risperidone treatment between May 2017 and May 2018 in Beijing Anding Hospital was performed in this study, and all clinical and demographic data were collected from the electronic health records. Each patient had two TDM results. One was the initial TDM value, which was the risperidone active moiety concentration measured within 5–14 days after the first administration. The other was the next TDM value that was closest to the initial TDM, which was set as the target variable. Influencing factors were screened from the data set as important variables to establish prediction model. Furthermore, 210 patients treated with risperidone between March 1, 2019, and May 31, 2019, in Beijing Anding Hospital were adopted as validation to verify the prediction ability of the model. The study was approved by Beijing Anding Hospital ethics committee (2020 NO.101) and compliance with the Helsinki Declaration and its revisions.

The inclusion criteria were (1) patients aged between 18 and 60 years; (2) patients treated with risperidone; (3) for variables that need to calculate the rate of change, the time limit was set as 1 week, and data were considered missing if the interval was more than 1 week. The exclusion criteria were (1) samples with risperidone and 9-hydroxyrisperidone concentrations below the lower limit of quantitation 2.5 ng/mL were deleted; (2) samples without risperidone records in long-term medical orders, samples collected TDM results before using risperidone, and those using risperidone continuously over 1 year were deleted.

As we aimed to study short-term risperidone exposure, the hospital stays of selected samples were set to be ≤ 3 months. Weight and height were measured through standard procedures, and body mass index (BMI) was calculated as weight (kg) divided by height squared (m^2^). A blood sample was taken at trough concentration and collected at 7 a.m. (after overnight fasting) after 5 days of continuous medication of risperidone. Serum concentration of the active moiety was measured using Applied Biosystems API 4000 QTrap with electrospray ionization (ESI), and Thermo Fisher U3000 high-performance liquid chromatograph (HPLC) (doi: 10.1002/bmc.4209). The analytes were extracted from serum samples automatically preconcentrated and purified by C_8_ solid-phase extraction cartridges, then chromatographed on an Xbridge™ C_18_ column (3.5 μm, 100 × 2.1 mm) thermostated at 30°C with a mobile phase at a flow rate of 0.3 mL/min, mobile phase A consisting of 0.1% formic acid in water and mobile phase B consisting of 0.1% formic acid in methanol and five microliter of extract were in a system kept at 50°C. Tandem mass spectrometric detection was carried out using positive ESI with an ionization voltage of 3,200 V, desolvation temperature of 500°C, cone of 40 L/h and while operating in the multiple reaction monitoring (MRM) mode. Sample storage at 4°C for 3 days was found stable. The linear range of both risperidone and 9-hydroxyrisperidone was 2.5–200 ng/ml. Intra- and inter-day precision and accuracy were evaluated by analyzing five replicates at a low limit of quantification and quality control samples at low, medium, and high concentrations. Intra- and inter-day precision for all analytes was between 1.1 and 8.2%; method accuracy was between 6.6 and 7.6%. The results were analyzed by Analyst 1.6.1 software. All data about risperidone usage, including the initial TDM value, dosage, and interval between two TDMs, were documented.

### Variable Selection

Multiple variables may influence the TDM results, including demographic data [age, height, weight, and body mass index (BMI)], risperidone information (dosage and concentration), drug combinations, other therapy [modified electroconvulsive therapy (MECT)], and assay index. Specifically, we involved 26 variables relating to the risperidone TDM value for modeling. Drug combinations include 5-hydroxytryptamine (5-HT), CYP2D6 enzyme inducers (carbamazepine, phenytoin sodium, phenobarbital, and rifampicin), citalopram, sertraline, and haloperidol. Some infrequently used other_ CYP2D6 enzyme inhibitors include quinidine, duloxetine, amiodarone, amitriptyline, bupropion, chlorpheniramine, chlorpromazine, clomipramine, diphenhydramine, and doxepine. Data of assay index include indexes of renal function, liver function, prolactin (PRL), and routine blood test.

**Figure 1:**
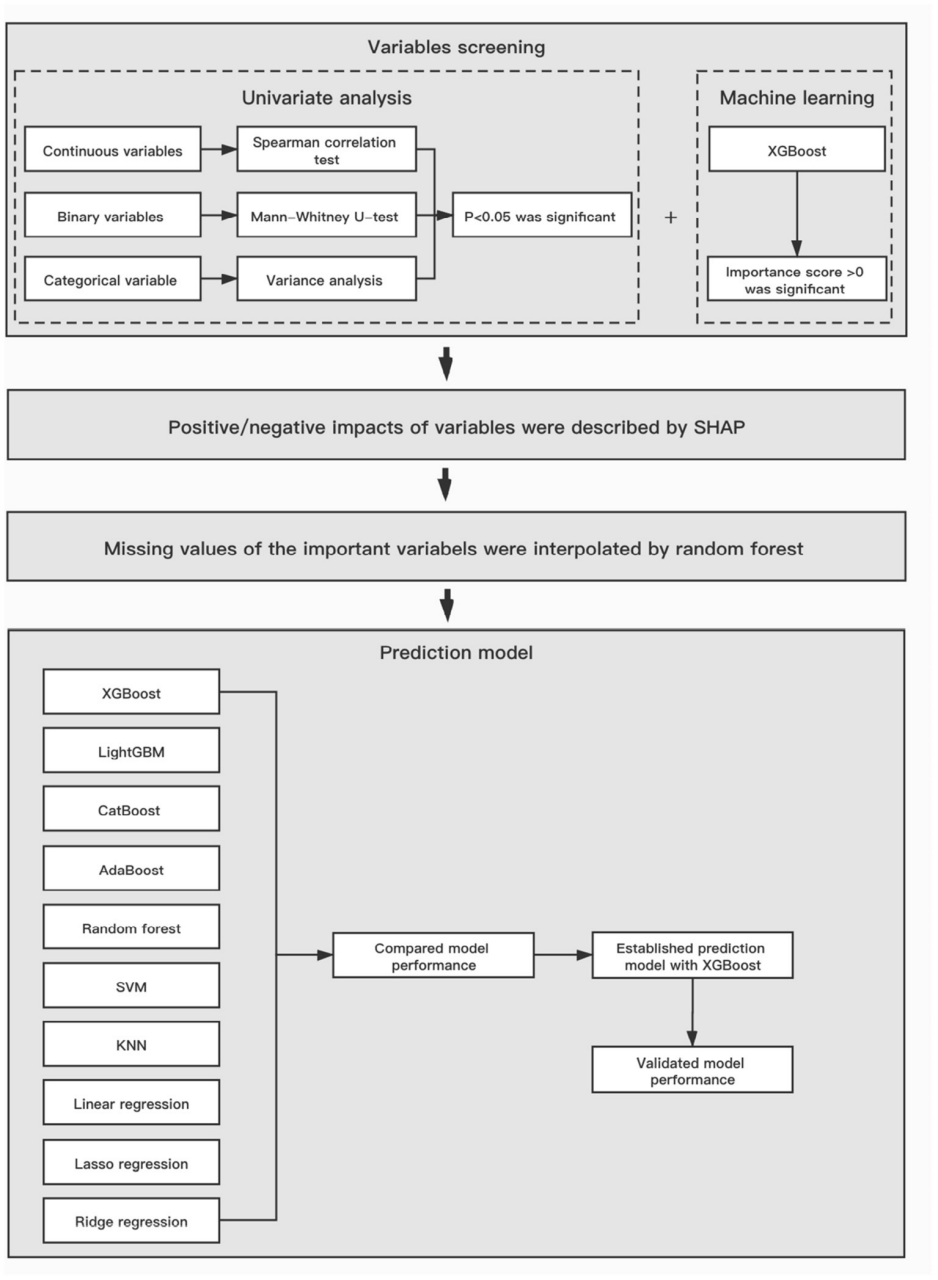
The workflow of data analysis and model establishment. XGBoost, Extreme Gradient Boosting; SVM, support vector machine; KNN, k-Nearest Neighbor.

The workflow of data analysis is illustrated in Figure 1. First, univariate analysis was implemented on all data to screen the significant variables, *p* < 0.05 was considered statistically significant. Additionally, all variables were screened using XGBoost, which calculated their importance scores and extracted the ones of which having score >0 to avoid the interference of a large number of irrelevant factors in the data set. Then, the SHapley Additive exPlanations (SHAP) method, a recent approach to convert the nonlinear XGBoost model into cumulative effects of all variable attributions, was performed to better interpret their positive or negative impacts on the final prediction (20).

### Model Establishment

According to the 8:2 proportional division, the total study population was divided into derivation and test cohorts. As depicted in Figure 1, we used 10 different algorithms for modeling to compare the prediction abilities on the next risperidone TDM, including XGBoost, LightGBM, CatBoost, AdaBoost, Random Forest, support vector machine (SVM), lasso regression, ridge regression, linear regression, and k-nearest neighbor (KNN) (21). To measure and compare model performance, *R*^2^, mean square error (MSE), root mean square error (RMSE), mean absolute error (MAE), and the accuracy of the predicted TDM within ±30% of the actual TDM were used as metrics. Algorithms with higher metric values were selected for the prediction model of next risperidone TDM.

### Statistical Analysis

For continuous variables, the Spearman correlation test was used for analysis. For binary variables, Mann–Whitney *U*-test was used for analysis. Categorical variables with an imbalance ratio <100:1 or variables with a missing rate >80% were excluded in the statistical analysis. The remaining missing values in the data set were interpolated by the random forest method. Data analysis was conducted using the Statistical Package for the Social Sciences software version 23.0, and Python 3.6.8.

## Results

### Baseline Information

From May 2017 to May 2018 in Beijing Anding Hospital, a total of 983 eligible patients were enrolled in this study. The baseline information of study population is shown in Table 1. It can be seen that the median age of the patients was 37.0 and the Inter Quartile Range (IQR) was 29.0~50.0, female were 53.8% of the total. Patients' median BMI was 24.0 kg/m^2^ (IQR 20.9~27.3 kg/m^2^). The median value of the initial risperidone TDM was 24.9 ng/ml (IQR 17.1~36.3 ng/ml), median dose of risperidone was both 4.0 mg (IQR 3.0~5.0 mg), and median TDM interval of risperidone was 7.0 days (IQR 6.0~9.0 days).

**Table 1:** Baseline characteristics of study population.

**Category**	**Variable**	**Entire data set (*n* = 983)**
Target variable	The next TDM, ng/ml, n (%)	32.9 (23.3–46.5)
Condition of risperidone use	The initial TDM, ng/ml, median (IQR)	24.9 (17.1–36.3)
	Risperidone dose, mg, median (IQR)	4.0 (3.0–5.0)
	TDM interval, days, median (IQR)	7 (6–9)
Demographic information	Age, year, median (IQR)	37.0 (29.0–50.0)
	Sex, n (%)	
	Male	454.0 (46.2%)
	Female	529.0 (53.8%)
	Height, cm, median (IQR)	165.0 (160.0–172.0)
	Weight, kg, median (IQR)	66.0 (57.0–76.0)
	BMI, kg/m^2^, median (IQR)	24.0 (20.9–27.3)
Combination	5-HT, n (%)	47.0 (4.8%)
	CYP2D6 enzyme inducers	1.0 (0.1%)
	Other_CYP2D6 enzyme inhibitors	250.0 (25.4%)
	Citalopram	13.0 (1.3%)
	Haloperidol	13.0 (1.3%)
	Sertraline	13.0 (1.3%)
Assay index	PLT, 10^9^/L, median (IQR)	244.0 (207.0–290.0)
	WBC, 10^12^/L, median (IQR)	6.5 (5.3–7.9)
	RBC, 10^9^/L, median (IQR)	4.6 (4.2–4.9)
	BUN, mmol/L, median (IQR)	3.7 (3.0–4.5)
	Cr, μmol/L, median (IQR)	60.0 (52.0–70.0)
	Ccr, ml/min, median (IQR)	123.8 (101.9–148.7)
	AST, U/L, median (IQR)	16.4 (13.4–22.1)
	ALT, U/L, median (IQR)	17.8 (11.9–27.1)
	PRL, μg/L, median (IQR)	62.8 (38.0–107.7)
	Last PRL, μg/L, median (IQR)	40.7 (28.3–73.4)
	PRL_change rate, %, median (IQR)	0.3 (0.1–1.0)
Other therapy	Using MECT within 1 week before TDM	288.0 (29.3%)

*IQR, inter quartile range; TDM, therapeutic drug monitoring; BMI, body mass index; PLT, platelet; RBC, red blood cells; WBC, white blood cells; BUN, blood urea nitrogen; Cr, serum creatinine; Ccr, creatinine clearance rate; ALT, alanine transaminase; AST, aspartate transaminase; PRL, prolactin; MECT, modified electroconvulsive therapy*.

### Selected Variables

In the univariate analysis, some variables were excluded because of extremely uneven distribution or lots of missing values, such as 5-HT, CYP2D6 inducers, citalopram, sertraline, the last PRL, and the change rate of PRL. The statistical results of the remaining 20 variables are shown in Table 2, of which six significant variables had *p* < 0.05, including risperidone dose, the initial TDM value, age, PLT, WBC, and MECT. Furthermore, the screening results of XGBoost present the contribution of each variable by their importance score, shown in Table 3. A higher importance score indicates greater significance of variables relating to next risperidone TDM value. There were 18 variables having importance score >0, which were selected to proceed to the further analysis. The most important variable relating next risperidone TDM was the initial TDM value with an importance score of 0.2522. Ultimately, 16 variables with statistical significance in univariate analysis or those with importance score >0 in XGBoost screening were selected, associated with clinical prior knowledge, we excluded height, haloperidol, sex, and other_CYP2D6 inhibitors. Specifically, height, weight, and BMI were all important influencing factors, but there was a quantitative relationship among them. To simplify calculation, two variables, namely, weight and BMI, were retained. In addition, haloperidol was generally used to control agitation symptoms in patients, which was a short-term and temporary medication. There was no drug–drug interaction between haloperidol and risperidone; thus, it is difficult to explain why it causes risperidone concentration to increase. Other_CYP2D6 inhibitors were excluded because of a limited number of medication cases and low frequency monitoring. Sex was excluded due to their importance score of 0, meaning very low impact on next risperidone TDM.

**Table 2:** Statistical results of univariate analysis.

**Variable**	**Statistics (r/U/F)**	***p*-value**
Risperidone dose	0.360	<0.001
The initial TDM	0.624	<0.001
TDM interval	0.041	0.203
Age	−0.113	<0.001
Height	0.005	0.865
Weight	0.023	0.469
BMI	0.024	0.456
PLT	0.073	0.024
WBC	0.106	<0.001
RBC	0.041	0.207
BUN	0.008	0.798
Cr	0.039	0.002
Ccr	0.048	0.141
PRL	0.061	0.116
AST	−0.020	0.536
ALT	0.020	0.541
Sex	113229.5	0.123
MECT	88230.0	0.003
Haloperidol	84488.0	0.158
Other_CYP2D6 enzyme inhibitors	86395.0	0.177

*TDM, therapeutic drug monitoring; BMI, body mass index; PLT, platelet; RBC, red blood cells; WBC, white blood cells; BUN, blood urea nitrogen; Cr, serum creatinine; Ccr, creatinine clearance rate; ALT, alanine transaminase; AST, aspartate transaminase; PRL, prolactin; MECT, modified electroconvulsive therapy*.

**Table 3:** The importance score of variables in XGBoost.

**Variables**	**Importance score**
The initial TDM	0.2522
Risperidone dose	0.0616
Ccr	0.0598
Weight	0.0592
BUN	0.057
WBC	0.0527
Height	0.0522
ALT	0.0512
PLT	0.0457
MECT	0.0394
AST	0.0394
Haloperidol	0.0381
RBC	0.0367
Cr	0.0366
PRL	0.0341
BMI	0.0327
Age	0.0279
TDM interval	0.0235
Sex	0
Other_CYP2D6 enzyme inhibitors	0

*TDM, therapeutic drug monitoring; BMI, body mass index; PLT, platelet; RBC, red blood cells; WBC, white blood cells; BUN, blood urea nitrogen; Cr, serum creatinine; Ccr, creatinine clearance rate; ALT, alanine transaminase; AST, aspartate transaminase; PRL, prolactin; MECT, modified electroconvulsive therapy*.

**Figure 2:**
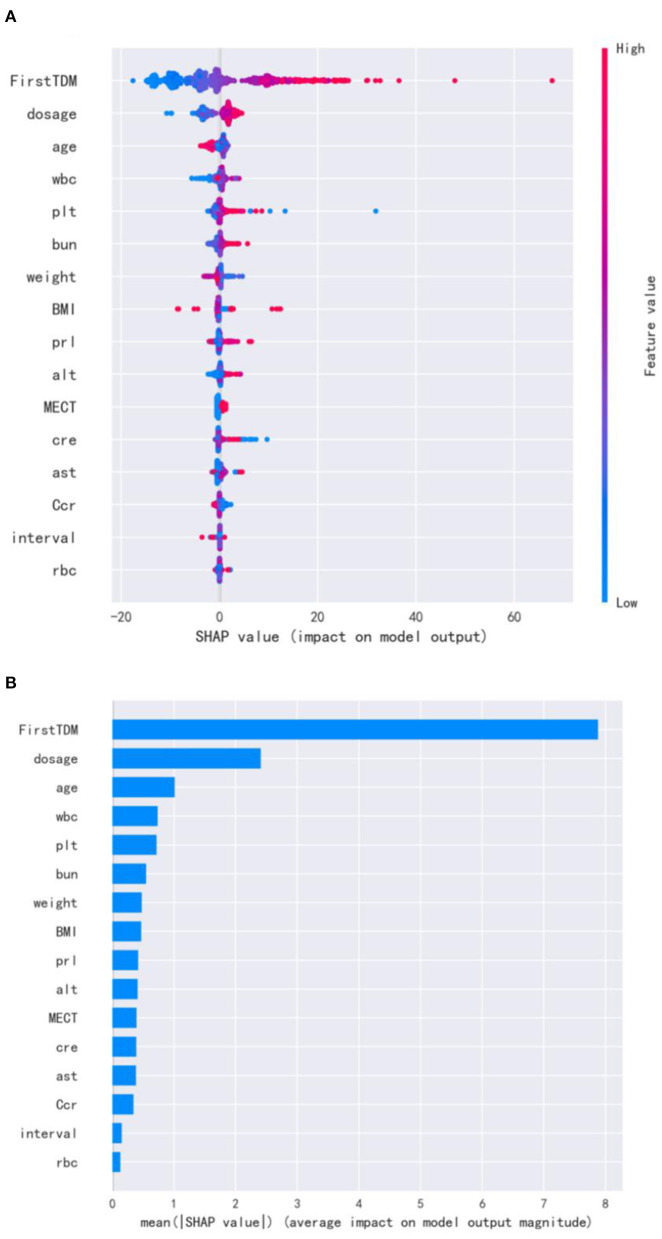
SHAP for important variables affecting the next risperidone TDM. **(A)** visualization of the variable impacts. The dot color is redder when the feature value gets higher and bluer when the feature value gets lower. When the SHAP value gets higher, the impact of the variable on model output is larger. **(B)** Ranking of the mean absolute SHAP values. The larger mean absolute SHAP value indicates the stronger correlation.

For the visualization of the variable importance, we used SHAP to display how these variables affected the next risperidone TDM value (Figure 2). The correlations of these variables with the next risperidone TDM value were compared by calculating the mean absolute SHAP value of each variable. The larger mean absolute SHAP value indicates the stronger correlation. The variables showing positive correlations with model prediction outcome included the initial TDM value, risperidone dose, WBC, PLT, BUN, PRL, alanine transaminase (ALT), and MECT, strongest to weakest in a descending order. Those negatively correlated with model prediction outcome included age, weight, BMI, creatinine clearance rate (Ccr), strongest to weakest in descending order. The distinct influencing direction of creatinine (Cr), aspartate transaminase (AST), TDM interval, and RBC cannot be seen.

### Model Establishment and Validation

After variable selection, random forest was used to interpolate the missing values; see Supplementary Table S1. Subsequently, based on the selected variables, 10 models (XGBoost, LightGBM, CatBoost, AdaBoost, Random Forest, SVM, KNN, linear regression, lasso regression, and ridge regression) predicting the next risperidone TDM were established. To further consolidate the performance of models, six-fold cross-validation was used to present the prediction results of 10 algorithms in a test cohort (Supplementary Table S2). The final model performance in the test cohort (N = 197) was illustrated in Table 4. The XGBoost model had the highest *R*^2^ (0.512), demonstrating good model fit. Besides this, in the XGBoost model, the accuracy of the predicted TDM within ±30% of the actual TDM was 54.82%, the highest of the 10 models. Values of MAE, MSE, and RMSE in the XGBoost model were 10.97, 198.55, and 14.09, respectively, the low value representing good fit of the model. Thus, XGBoost had the most prominent model performance and was chosen to be applied for the prediction model.

**Table 4:** The prediction results of 10 algorithms in the test cohort.

**Model**	**R^**2**^**	**MAE**	**MSE**	**RMSE**	**Accuracy of the predicted TDM within ±30% of the actual TDM**
**XGBoost**	**0.512**	**10.97**	**198.55**	**14.09**	**54.82%**
LightGBM	0.269	12.85	297.52	17.25	47.72%
CatBoost	0.403	11.55	242.88	15.58	51.78%
AdaBoost	0.344	13.13	267.07	16.34	45.18%
Random forest	0.487	11.17	208.69	14.45	52.82%
SVM	0.486	10.60	209.39	14.47	53.81%
KNN	0.181	13.76	333.38	18.26	48.22%
Linear regression	0.482	11.18	210.98	14.53	54.31%
Lasso regression	0.482	11.19	210.97	14.52	54.31%
Ridge regression	0.482	11.19	210.99	14.53	54.31%

*XGBoost, Extreme Gradient Boosting; SVM, support vector machine; KNN, k-Nearest Neighbor; MSE, Mean Square Error; RMSE, Root Mean Square Error; MAE, Mean Absolute Error. Bold values mean the best prediction performance among ten models*.

Furthermore, the model performance was validated in a validation cohort (N = 210). In Table 5, *R*^2^ decreased but the fluctuation was within the normal range, indicating that the fitting ability of the model decreased, probably due to different data sources. Meanwhile, the accuracy of the predicted TDM within ±30% of the actual TDM increased to 60.95%, proving that the actual prediction effect is good, and the model can predict risperidone TDM well.

**Table 5:** The prediction results in validation cohort.

**R^**2**^**	**MAE**	**MSE**	**RMSE**	**Accuracy of the predicted TDM within ±30% of the actual TDM**
0.374	12.07	324.15	18.00	60.95%

*MSE, Mean Square Error; RMSE, Root Mean Square Error; MAE, Mean Absolute Error*.

## Discussion

Risperidone is the most commonly prescribed antipsychotic medication to treat approximately 31.1% patients with schizophrenia in China (22, 23). Of late, researchers commonly used population pharmacokinetic (PopPK) models to study individualized drug regimens, frequently with the nonlinear mixed-effects modeling (NONMEM) analysis (22, 24, 25). Some PopPK models were applied to analyze risperidone and its active moiety concentrations. However, PopPK models were commonly performed under restricted conditions, and its parameters were generally affected by the wide inter-individual differences and the interactions of concomitant medications (24, 25).

In the present work, the prediction model was ultimately established with XGBoost, one of the machine learning algorithms, which is a leading-edge method based on the decision tree principle and the effective upgrade of the gradient boosted decision tree (GBDT) algorithm (21). It integrates a series of decision trees to achieve the classification or regression goals. Its major advantages include (1) rapid and effective, (2) supporting parallel computing and column sampling to prevent overfitting, (3) incorporating regularization to control the model complexity, (4) setting built-in rules to deal with missing values, (5) setting built-in cross-validation, (6) promising robustness to highly correlated variables and sparse matrix, and (7) ability to select important variables (21). Machine learning is suitable to process a large volume of real-world data, deal with missing values and high-dimensional data, and capture complicated relationships between variables, especially for retrospective studies. Therefore, we attempted to identify the model predictors via the XGBoost method for model stability and prediction precision. Hence, the selected variables were of great significance to the next risperidone TDM value. There was another study of 407 patients predicting vancomycin trough concentration using an ensemble machine learning model with five algorithms (XGBoost, GBRT, Bagging, ExtraTree, and decision tree), achieving a prediction accuracy (predicted trough concentration within ±30% of the actual trough concentration) of 51.22% in a test cohort (26). In comparison, our study had better performance (accuracy of the predicted TDM within ±30% of the actual TDM is 54.82%) in the test cohort and also larger sample size, which led to a more mature model for application and reference. Normally, with increasing samples involved, the model performance will get better, which is a property of machine learning models.

In the aspect of influencing variables to the next risperidone TDM value, certain variables, including age, weight, BMI, and dosage were frequently selected as basic predictors in PopPK model. The initial TDM value was the most prominent influencing variable found in the present study, if we obtained the initial risperidone TDM information and then predicted the next TDM value, the drug regimen could be adjusted based on the prediction model. Besides this, because 9-hydroxyrisperidone is metabolized primarily in the liver, ALT and AST reflect liver function and associate tightly with risperidone concentrations (4, 8, 9). In addition, the main excretion pathway is renal metabolism, and a former study demonstrates that around 70% of risperidone is removed through urine (16). BUN, Cr, and Ccr are indicators of renal function, which are also included in the prediction model according to our results. It is proven in a long-term study that risperidone treatment induced PRL increase and hyperprolactinemia (27). Serum concentration of 9-hydroxyrisperidone was predominantly related to increased PRL levels, especially in autistic patients with hyperprolactinemia during risperidone treatment (28). In our results, PRL was selected via XGBoost as an important variable to predict the next risperidone TDM value, which could be deemed as an *a posteriori* predictor. Additionally, drug combinations (CYP2D6 enzyme inducers and others) were also crucial variables we took into consideration initially. CYP2C19, CYP3A5, SLCO1B1, UGT1A1, ABCB11, and ADH7 are all involved in the metabolism of risperidone into 9-hydroxyrisperidone. Inhibitors or inducers of these liver drug enzymes and transporters can affect risperidone and 9-hydroxyrisperidone concentrations (29–31). Some showing drug–drug interactions in previous research, such as high-dose sertraline, may elevate the serum concentration of risperidone (32, 33). However, these known drugs were not selected into the ultimate list of important variables, some CYP2D6 inhibitors had limited numbers of medication cases and missed opportunities to monitor the changes in concentration (low frequency monitoring) when briefly combined with risperidone, creating an illusion that these inhibitors did not lead to the increase of risperidone concentration. These variables may be deeply studied in future research with a large number of samples. Notably, we included MECT in initial variable screening as well, which was rarely seen in other similar studies. As an efficient method for treating drug-resistant schizophrenia, MECT may prompt some patients more sensitive to risperidone (33). Based on current knowledge, it is difficult to explain the relationship between some newfound indicators (WBC, RBC, and PLT) and the next risperidone TDM value. This may result from the scarcity of positive samples, immature application of the machine learning algorithm, or these uncommon indicators were possibly confounding factors. It necessitates further research to explore whether these indicators influenced the sample's blood status or they were noises affected by samples' heterogeneity.

To date, the viewpoints about impacts of CYP2D6 genotypes on risperidone and its active moiety levels remain inconsistent. Some believe that the polymorphism of CYP2D6^*^10 was related to steady-state plasma concentration of risperidone, demonstrated by research on 443 Indian patients with schizophrenia (34). Conversely, some others stated that there existed no correlation between CYP2D6 genotypes and risperidone. A study based on 97 Thai children and adolescents with autism spectrum disorder suggested that CYP2D6 genotypes were independent of the risperidone plasma concentrations or the total active moiety levels (35). Herein, due to the limited research conditions, the exploration of cytochrome enzyme genotypes were not involved in our study, which may be considered in further research.

One limitation of the present work was limited positive samples. It is easier for machine learning to learn groups with larger sample sizes. Furthermore, some potent CYP2D6 enzyme inhibitors were known to elevate the active moiety of risperidone, such as paroxetine (36). However, the data we collected from the clinical center demonstrated that the common combined drugs were medium-strength inhibitors with less toxicity, such as duloxetine and bupropion. The reasons for CYP2D6 enzyme inhibitors not selecting into the final model include limited cases and the low frequency of data collection, and positive TDM results were not easily captured after using risperidone. Because certain uncommon variables were selected into the model instead of some recognized variables (CYP2D6 enzyme inhibitors), we concluded that data collected with high frequency could be better studied by machine learning. Henceforth, it is necessary to constantly optimize the model using a larger sample size and collecting important variables more frequently. Third, the samples we used for validation were from the same medical center; in the future, an external validation from different centers could be conducted in the analysis to verify the model generalization. Another limitation was that, due to the limited research conditions, pharmacogenetic data of patients were not included in the analysis in this study, such as the genotype of D_2_ receptor (the pharmacodynamic target of risperidone), and the genotype of CYP2D6 (the main metabolic enzyme of risperidone); therefore, the study was confined to the drug concentration level.

In conclusion, we selected 16 predictors and constructed a XGBoost model to predict the next active moiety concentration of risperidone based on the initial TDM value. The prediction model is proven to have promising accuracy (accuracy of the predicted TDM within ±30% of the actual TDM is 54.82% in test cohort, and 60.95% in validation cohort). To our knowledge, this study represents the first prediction model of risperidone active moiety concentration based on initial TDM via machine learning methods, which is meaningful for the rational risperidone regimen on individualized level, and the modeling process can potentially be referenced to other antipsychotic drugs in the future.

## Data Availability Statement

The raw data supporting the conclusions of this article will be made available by the authors, without undue reservation.

## Ethics Statement

The studies involving human participants were reviewed and approved by Ethics Committee of Beijing Anding Hospital. The Ethics Committee waived the requirement of written informed consent for participation.

## Author Contributions

WG, ZY, XL, and YZ designed the study. YG and FG guided the study. PY, ZW, and WS were responsible for statistical analysis. XH wrote the manuscript. All authors contributed to data interpretation and read and approved the final manuscript for publishing.

## Funding

This research was supported by Capital's funds for health improvement and research (2018-4-2124), Beijing municipal administration of hospitals incubating program (PZ2020031, PX2019069), and National Key Research and Development Program (2020YFC2005502, 2020YFC2005503).

## Conflict of Interest

ZY, PY, ZW, and FG are employed by Beijing Medicinovo Technology Co. Ltd. XH is employed by Dalian Medicinovo Technology Co. Ltd. The remaining authors declare that the research was conducted in the absence of any commercial or financial relationships that could be construed as a potential conflict of interest.

## Publisher's Note

All claims expressed in this article are solely those of the authors and do not necessarily represent those of their affiliated organizations, or those of the publisher, the editors and the reviewers. Any product that may be evaluated in this article, or claim that may be made by its manufacturer, is not guaranteed or endorsed by the publisher.
