# Supplementary material for: A Machine Learning Model to Predict Risperidone Active Moiety Concentration Based on Initial Therapeutic Drug Monitoring
Source: Front Psychiatry. 2021 Nov 18;12:711868. doi: 10.3389/fpsyt.2021.711868 (PMC8637165; doi:10.3389/fpsyt.2021.711868)
Supplement: Supplementary file 1 [file Table_1.DOCX]

**Supplementary**

**Table S1. Missing rates of the important variables**

| No. | Variables | Missing rates (%) |
| --- | --- | --- |
| 1 | The initial TDM | 0.00% |
| 2 | Risperidone dose | 0.00% |
| 3 | TDM interval | 0.00% |
| 4 | Age | 0.00% |
| 5 | Weight | 0.00% |
| 6 | BMI | 1.42% |
| 7 | PLT | 1.83% |
| 8 | WBC | 1.83% |
| 9 | RBC | 1.83% |
| 10 | BUN | 2.24% |
| 11 | Cr | 2.54% |
| 12 | Ccr | 2.54% |
| 13 | PRL | 32.66% |
| 14 | AST | 1.73% |
| 15 | ALT | 2.03% |
| 16 | MECT | 0.00% |

Abbreviations: TDM, therapeutic drug monitoring; BMI, body mass index; PLT, platelet; RBC, red blood cells; WBC, white blood cells; BUN, blood urea nitrogen; Cr, serum creatinine; Ccr, creatinine clearance rate; ALT, alanine transaminase; AST, aspartate transaminase; PRL, prolactin; MECT, modified electroconvulsive therapy.
