# Supplementary material for: A Machine Learning Model to Predict Risperidone Active Moiety Concentration Based on Initial Therapeutic Drug Monitoring
Source: Front Psychiatry. 2021 Nov 18;12:711868. doi: 10.3389/fpsyt.2021.711868 (PMC8637165; doi:10.3389/fpsyt.2021.711868)
Supplement: Supplementary file 2 [file Table_2.DOCX]

Table S2. The prediction results of ten algorithms in test cohort using 6-fold cross validation

| **Model** | **R^2^** | **MAE** | **MSE** | **RMSE** | **Accuracy of the predicted TDM within ±30% of the actual TDM** |
| --- | --- | --- | --- | --- | --- |
| XGBoost | 0.424±0.036 | 11.19±0.58 | 232.28±30.22 | 15.21±1.02 | 54.91±2.95% |
| LightGBM | 0.276±0.037 | 12.52±0.48 | 291.46±34.89 | 17.05 ± 1.03 | 51.61± 2.68% |
| CatBoost | 0.416±0.036 | 11.16±0.58 | 235.69±34.05 | 15.32±1.14 | 56.43±4.07% |
| AdaBoost | 0.316±0.065 | 13.0±0.71 | 275.17±37.28 | 16.56±1.14 | 46.87±2.76% |
| Random forest | 0.314±0.021 | 12.57±0.62 | 277.59±40.44 | 16.62±1.25 | 49.74±2.98% |
| SVM | 0.423±0.045 | 10.61±0.64 | 232.74±31.9 | 15.22±1.08 | 59.98±3.75% |
| KNN | 0.165±0.027 | 13.84±0.89 | 338.22±53.27 | 18.34±1.49 | 47.97±2.58% |
| Linear regression | 0.44±0.055 | 10.92±0.48 | 225.0±27.16 | 14.98±0.92 | 55.84±3.76% |
| Lasso regression | 0.44±0.055 | 10.92±0.48 | 224.97±27.2 | 14.98±0.92 | 56.09±3.84% |
| Ridge regression | 0.44±0.055 | 10.91±0.48 | 224.97±27.3 | 14.98±0.93 | 56.01±3.42% |
